# The Pros and cons of balloon dilation in totally ultrasound-guided percutaneous Nephrolithotomy

**DOI:** 10.1186/s12894-020-00654-x

**Published:** 2020-07-01

**Authors:** Wei Jin, Yan Song, Xiang Fei

**Affiliations:** Urology Department, Sheng Jing Hospital of China Medical University, Shenyang, 110000 China

**Keywords:** Percutaneous nephrolithotomy, Renal stone, Urolithiasis, Ultrasound

## Abstract

**Background:**

To evaluate the feasibility and safety of balloon dilation (BD) in totally ultrasound-guided percutaneous nephrolithotomy (PCNL).

**Methods:**

The data of 95 patients underwent BD were collected in this retrospective study between August 2016 and December 2018. During the same period, telescopic metal dilation was used in 1161 patients. Ninety five patients were selected as the control group and matched at a 1:1 ratio to index balloon dilation (BD) cases in regards to Guy’s stone score, age, sex, BMI, degree of hydronephrosis and stone area. Peri-operative data were compared between the two groups.

**Results:**

Total operative time was significantly shorter in the BD group (62.2 ± 22.4 min vs. 70.2 ± 25.8 min, *p* = 0.024). Tract establishment time was significantly shorter in the BD group (3.4 ± 1.8 min vs. 4.3 ± 2.3 min, *p* < 0.001). The success rate of tract dilation by first attempt was higher in the TMD group compared with that of BD group; however the difference was not statistically significant. There was no significant difference between groups with regards to complication and stone-free rates. The cost of PCNL in the BD group was significantly higher than that of the TMD group (US $4831.4 ± 1114.8 vs. US $4328.4 ± 975.7, *p* = 0.012). Subsequent analysis revealed that mild or no hydronephrosis were risk factor for failure of balloon dilation under ultrasound.

**Conclusions:**

BD has acceptable complication and stone free rates compared with those in TMD; however, BD under ultrasound is not suggested for stone cases without hydronephrosis.

## Background

Percutaneous nephrolithotomy (PCNL) was introduced by Fernstrom and Johansson in 1976 [1]. It has been suggested as the optimal treatment method for patients with kidney stones with the size over 2 cm or staghorn stones [2]. Balloon dilation (BD), telescopic metal dilation (TMD), Amplatz semi-rigid dilation, or a single-step(one shot) dilation was all used as tract establishment methods [3–5]. BD has been considered to be safer and more effective; it has widely been used in X-ray guided PCNL [6]. Previous reports have proved that BD is associated with less bleeding and less renal damage than other dilation methods [7]. However, X-ray guided PCNL is associated with radiation hazard to both the patients and intraoperative personnel [8].

Ultrasound-guided nephrolithotomy is a low cost percutaneous puncture technique; the success rates are high in well-trained urologists [9]. The safety and efficacy of PCNL solely guided by ultrasound has been well studied and reported [10, 11]. However, Amplatz and TMD was mostly performed in the previously reported ultrasound-guided PCNL,and the two-step method was reported to reduce the complication while establishing the working tract [11]; the safety and efficacy of ultrasound-guided balloon dilation has not been well studied. Herein, we aimed to evaluate the outcome of ultrasound–guided PCNL using BD and compare the outcome with that of PCNL using TMD.

## Methods

### Study design

The data of patients underwent PCNL with kidney stones over 2.0 cm, with multiple or staghorn stones were collected in this retrospective study.Patients with chronic renal failure, congenital abnormalities of the kidney (horseshoe kidney etc.), and solitary kidney were excluded.

Between August 2016 and December 2018, 95 patients who had kidney stones were treated in our hospital by ultrasound-guided PCNL using balloon dilation. CT was used in all patients for evaluation of the stone. Patients’ demographic data,such as age, sex, body mass index (BMI), size, number and location of the stone(s),past history of kidney operation were recorded. Preoperative laboratory tests included routine urine analyses and cultures,serum creatinine and blood routine, coagulation tests,. All patients signed an informed consent form before operation. The study was approved by the ShengJing hospital ethics committees (NO.2015PS266K). Authors had no access to information that could identify individual participants during or after data collection.

During the same period, Ultrasound-guided PCNL was performed in 1161 patients using telescopic metal dilation. From this cohort, we selected 95 patients as the control group. The 95 patients were matched at a 1:1 ratio to index balloon dilation (BD) cases with respect to Guy’s stone score which works as an independent predictive factor for complications and stone-free rate, as well as age, sex, BMI, degree of hydronephrosis and stone area. When more than one possible matches were available, controls were labeled with a random number generator within Excel (Microsoft Corp, Redmond, WA, USA) and PCNL data corresponding to the lowest random numbers assigned were selected as controls. Both of the procedures was the standard of care at the time and whether patients would receive PCNL using balloon dilation or telescopic metal dilation depended on the preference of the surgeon and availability of the equipment at the operation time. Operative data such as stone free rate, complication rate, cost, need for auxiliary treatment and postoperative hospital stay were evaluated.

Ultrasonographic guidance was used in all steps of the procedure during PCNL; X-ray was not used in this study.

### Intervention

After anesthesia, patients were first placed in the lithotomy position, and then the ureteral catheter was placed and secured. PCNL was performed after patients were placed in the prone or lateral position. A colored-Doppler ultrasound system with a 3.5-MHz transducer (Hitachi Aloka, Tokyo, Japan) was used. Under ultrasound guidance, an 18-gauge coaxial needle was targeted and introduced into the most convex point of the target calyx. In the case of no or mild hydronephrosis, saline was injected through the ureteral catheter to help the ballooning of the renal calyx. After puncture, the obturator was removed and a 5-mL syringe was attached to the needle to observe the recovery of the injected saline from the ureteral catheter which helped to confirm successful access to the renal calyx. J-tipped 0.038-in. guidewire was inserted after removal of the stylet, and the length of the needle from the skin to renal calyx was measured and marked to ensure that the length of the dilator was equal to that length [11].

### TMD group

Establishment of the working channel was done by using a previously described two-step method [11]. After dilation, a 24-Fr Alken sheath was positioned and a 20.8-Fr rigid nephroscope (Richard Wolf) was introduced into the renal calyx. Operation was performed by using a Swiss LithoClast®device.

### BD group

**Fig. 1:**
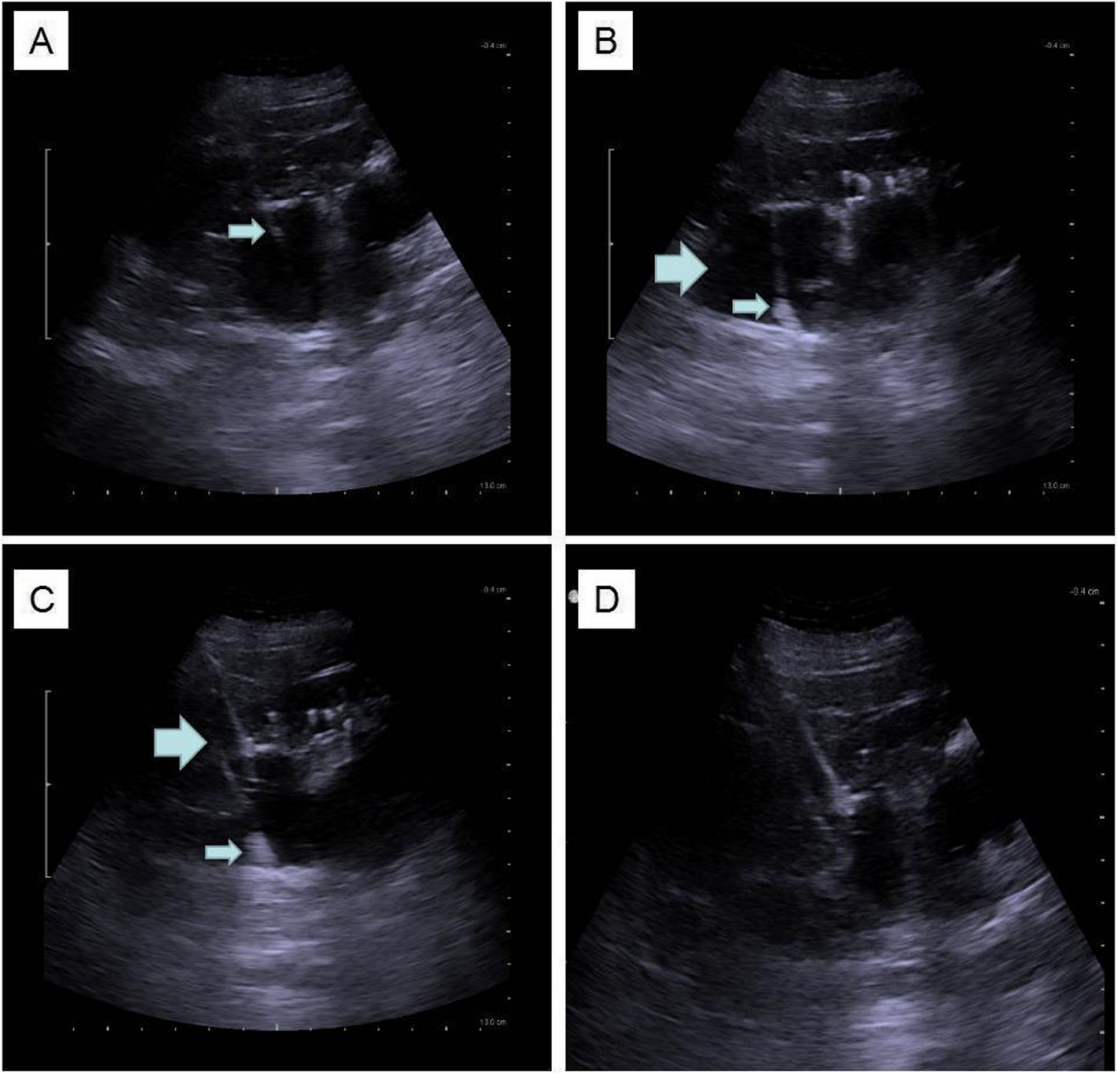
**a** Location of needle was confirmed, needle appeared as the bright “line” under ultrasound(small arrow). **b** Under the monitoring of ultrasound,location of guidewire was confirmed .Small arrow showed the soft tip of the guidewire coiled in the calyx and big arrows showed the dilated calyx. **c** Under the monitoring of ultrasound,location of balloon was confirmed; then the balloon was inflated with 25 atm for 1 min(big arrow). The soft tip of the guidewire was coiled in the calyx(small arrow) . **d** Location of working sheath was confirmed under ultrasound, the dilated calyx collapsed after urine flew out through the sheath

Using a stiff guidewire, the Balloon (30-Fr, BD Company, USA) was inflated up to 30 atm for 1 min. The inflation of the balloon was confirmed by ultrasound. First, a 6-Fr fascial dilator was inserted along the guidewire to pre-dilate the tract which can facilitate the insertion of the balloon dilator; the length of the balloon dilator between the skin and the renal calyx was equal to the dilation depth which was measured and marked by the 6-Fr fascial dilator. The location of balloon was confirmed under ultrasound. The 30-Fr sheath was passed over the inflated balloon and the balloon was then removed. The sheath was kept in place as the working channel (Fig. 1). Then, Calculus disintegration was performed as described above.

### Postoperative evaluation and follow-up

On the first postoperative day, hematocrit levels were assessed, Low dose CT scan was performed to evaluate status of residual stones 48 h after operation routinely. Nephrostomy tubes were removed after CT scan if a second-look procedure was not needed. Second-look PCNL, ureteroscopy, and shock wave lithotripsy were considered as auxiliary treatments .

### Outcome measures

Patients’ baseline demographic data, Guy’s STONE grade [12] and operative data were compared between the two groups. Modified Clavien–Dindo classification of surgical complications was used to evaluate the complicate [13]. Operative time was defined as the time from the placement of the ureteral catheter, until the access tract was sealed or nephrostomy tube was placed. The absence of visible fragments on CT was defined as stone free status. Non-obstructive residual stones that were less than 4 mm in diameter,, and asymptomatic were defined as clinically insignificant residual fragments (CIRF).

### Statistical analysis

Data were analyzed by SPSS (version 22; IBM Corporation; Armonk, NY, USA). For statistical analysis, continuous variables were given as mean or median (interquartile range) when necessary. Categorical values were given in frequency or percentages. T test, Welch T test and Mann Whitney U tests were used for continuous variables. Chi-square test was used for categorical variables. A *p* value < 0.05 was accepted as statistically significant.

## Results

**Fig. 2:**
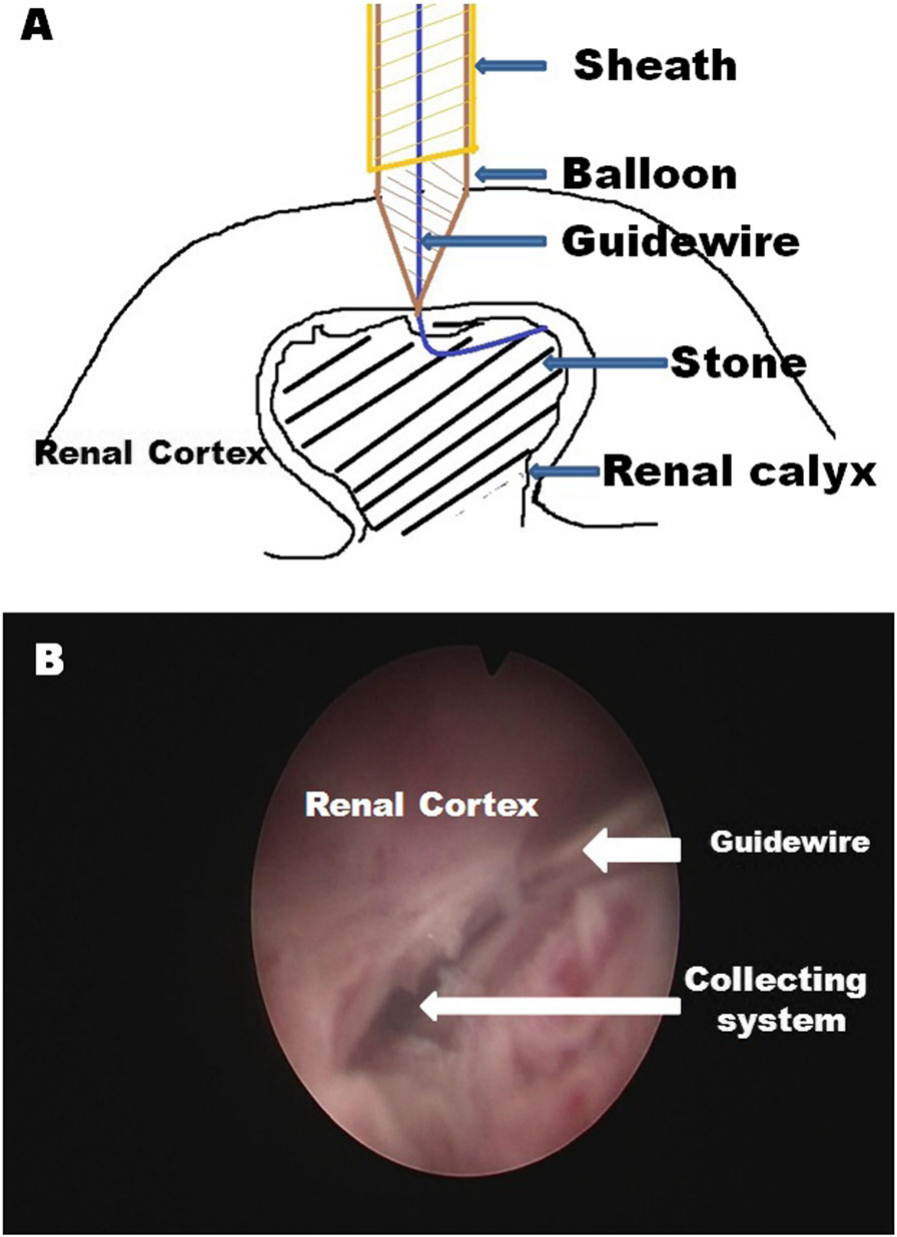
**a** Balloon dilation in case without hydronephrosis. **b** Short dilation inspected by nephroscope

One hundred ninety patients (95 in the BD group, 95 in the TMD group) were included in this study.Total operative time in the BD group was significantly shorter (62.2 ± 22.4 min vs. 70.2 ± 25.8 min, *p* = 0.024). Tract establishment time in the BD group was significantly shorter (3.4 ± 1.8 min vs. 4.3 ± 2.3 min, *p* < 0.001). The success rate of tract dilation by first attempt was higher in the TMD group compared with that of BD group; however there was no significant difference. In the BD group (*n* = 95), BD was successful in 83(87.4%) at first attempt but failed in 12 cases (8 cases were kidney stone without hydronephrosis). In such cases of no hydronephrosis, the cone-shaped tip of the balloon could slip outside the renal calyx right after inflation, as there was no space between the renal calyx and the stone to hold the tip of the dilator(Fig. 2). However, BD was successful on the second attempt in 10 cases and the remaining 2 cases were converted to TMD. In the TMD group, TMD was successful in 91 patients (95.8%) at the first attempt; and the remaining 4 cases were all successfully dilated on the second attempt. The stone-free rate after first session of PCNL was 82.1% (78/95) for the BD group, which was higher when compared with 78.9% (75/95) for the TMD group; but the difference was not significant (*p* > 0.05). The need for ancillary procedures (12 cases) was similar between groups (5.3% in BD group vs. 7.4% in TMD group). Second-look PCNL was necessary in 5 patients (3 in BD group and 2 in TMD group). Ureteroscopy was performed in 4 patients (2 patients in each group). SWL was performed in 3 patients (1 in BD group and 2 in TMD group). The cost of PCNL was numerically higher in the BD group than that in the TMD group (US $4831.4 ± 1114.8 vs. US $4328.4 ± 975.7, *p* = 0.012). (Table 1).

**Table 1:** Intraoperative and Postoperative data according to patients’ group

	Group 1 (95) Balloon dilation	Group 2 (95) Telescopic metal dilation	*P* value
Anesthesia (General/ epidural)	11/95	12/95	0.828
Success rate of dilation by first attempt	83/95 (87.4%)	91/95 (95.8%)	0.054
Operation time(min) mean ± SD	62.2 ± 22.4	70.2 ± 25.8	0.024
Tract establishing time(min) mean ± SD	3.4 ± 1.8	4.3 ± 2.3	< 0.001
Number of tract Single/multiple	85/10	84/11	0.885
Access of calyx			0.575
Upper	25	27	
Middle	49	43	
Lower	21	25	
Hb drop(g/dl) mean ± SD	3.2 ± 2.8	3.1 ± 1.7	0.821
Stone free rate (N, %)	78/95 (82.1%)	75/95 (78.9%)	0.735
CIRF rate	10/95 (10.5%)	11/95 (11.5%)	0.863
auxiliary treatments	5/95 (5.3%)	7/95 (7.4%)	0.175
Hospital stay (day) mean ± SD	3.2 ± 1.1	3.4 ± 1.5	0.614
Cost(U.S dollar)	$4831.4 ± 1114.8	$4328.4 ± 975.7	0.012

Abbreviations *CIRF* Clinical insignificant residue fragments

There was no significant difference between groups in regards to Clavien grade I, II, or III complications (18.9% in BD group vs.20.0% in TMD group, *p* = 0.827; Table 3). Neither of the group had grade IV or V complications (sepsis shock, bleeding requiring nephrectomy, or death). The blood transfusion rate was comparable between groups (2 in BD group and 3 in TMD group). Postoperative fever (> 38 °C) occurred in 10 patients in the BD group and 10 patients in the TMD group,all the patients were successfully treated with antibiotics. Serious postoperative hematuria occurred in one patient after BD and two patients after TMD. They were treated with angio-embolization after failure to control the bleeding (Table 2).

**Table 2:** Complications of percutaneous nephrolithotomy classified according the modified Clavien system

	Group 1 (95) Balloon dilation	Group 2 (95) Telescopic metal dilation	*P* value
Grade 1			
Nephrostomy tube displacement	0	1	–
Transient fever < 38 °C	10	10	–
Grade 2			
bleeding requiring transfusion	2	3	–
Nonseptic infections requiring additional antibiotics	5	4	
Grade 3			
Grade 3 a			
Bleeding requiring embolization	1	2	
Grade 4	–	–	–
Grade 5	–	–	–
Overall (N, %)	18/95 (18.9%)	19/95 (20.0%)	0.827

Data are presented as n (%)

Subsequent analysis revealed that mild or no hydronephrosis were risk factor for failure of balloon dilation under ultrasound, while BMI, laterality, calyx of puncture, were not related to the success rate of balloon dilation. (Table 3).

**Table 3:** Analysis of risk factors for the failure of balloon dilation in ultrasound-guided PCNL

Variables	Number (95)	Success rate (85.6%) 83/95	*P* Value
BMI			0.085
≥ 30 kg/m2	44	38 (86.3%)	
< 30 kg/m2	51	45 (88.2%)	
Access of calyx			0.712
upper	25	21 (84.0%)	
middle	49	44 (89.8%)	
lower	21	18 (85.7%)	
Hydronephrosis degree			0.015
Mild or none	28	18 (64.3%)	
Moderate	46	44 (95.7%)	
Severe	21	21 (100.0%)	
Side of the Puncture			0.727
Left	48	41 (85.4%)	
Right	47	42 (89.4%)	

## Discussion

X-ray guidance is commonly used for PCNL. Given the recurrent nature of nephrolithiasis and high volume of patients in some stone center, cumulative exposure to radiation may be of significant concern to both patients and intraoperative personnel [14]. A prospective study done by Ortiz [15] demonstrated total radiation dose increases in proportion to BMI;which was caused by automatically increased radiation dose in over weight patients. Thomas Chi and his colleagues also demonstrated that radiation exposure dose increase with BMI [16]. Applying ultrasound guidance to PCNL for patients with higher BMI may potentially reduce radiation exposure for patients and staff. Other advantages of ultrasound over fluoroscopy include improved visualization of adjacent viscera, clearer delineation of the anterior and posterior calyces, detection of radiolucent stones and the avoidance of vascular injury with colored Doppler imaging [17].

The establishment of working tract is the critical step of PCNL. Previous reports have suggested that balloon dilation is safer and associated with less hemorrhagic complications [7–9]. However, according to recently published outcomes from the Clinical Research Office of the Endourological Society, more transfusions and significant hematocrit level declines were found in patients undergoing BD compared with those who underwent telescopic/serial dilation [18]. Tomaszewski and his colleage suggested that BD was associated with lower blood transfusion rate [19]. However, Wezel et al. found more complication in patients using BD [20]. Joel et al. [21] reported a 17% dilatation failure rate in 99 patients.using balloon dilation in X-ray guided PCNL. However, Osman et al. [22] reported a failure rate of less than 3.5% in a series of more than 300 patients using TMD. Most of the studies mentioned above were using X-ray guidance in performing PCNL. There are few studies concerning balloon dilation method in totally ultrasound-guided PCNL in the literature. Ren et al. compared the outcomes of PCNL using two tract dilation methods under unltrasound guidance and concluded that BD had a higher success rate of access creation and less blood loss [23].Zhou et al. suggested that BD was preferable for beginners because it was associated with less hemorrhage complication compared with that of Amplatz dilation [24]. However, we failed to find any significant difference between two groups regarding blood loss and complication in this study; The Hb drop was mainly caused by hemodilution. During the operation and after operation, approximately 2000 ml fluid was given to the patient.However, in cases of transfusion and embolization, the Hb drop was caused by hemorrhage, and usually the Hb drop was more than 10 g/dl. In this series, hemostatic coagulant was not used after completion of the procedure at working port removal,but we did use some hemostatic coagulant by IV right after the operation. The success rate of tract dilation by first attempt was somewhat lower in the BD group although the result was not statistically significant.

In the present study, the operative time in the BD group was shorter compared with that in the TMD group; and the stone-free rate in the BD group was higher (not statistically significant)after the first operation; these differences could be related to less tract dilation time and the softer and bigger working channel in the BD group(F30). Using a softer sheath allows for extraction of bigger stone fragments, which results in decreased lithotripsy time and number of stone fragments. The use of ureteroscope to make sure the puncture was a good additional step in the TMD group, but this may add to operative time already longer in this group.

The use of TMD or the Amplatz dilator cannot be monitored under ultrasound, which may lead to dislodgement of the guidewire, and perforation of collecting system during tract dilation. In order to overcome this drawback, we developed the two-step dilation method. By using a 16-Fr peel-away sheath, the ureteroscope could be introduced into the renal calyx to check the position of the puncture point, helping to avoid further damage to the kidney if the tract was not in the proper position. There were some major differences between the two dilation methods: first, each step of BD can be performed under ultrasound; second, BD simplifies the entire procedure of tract establishment and reduces potential trauma and the chance of guidwire dislodgement related to serial manipulation of the Amplatz dilator, especially for inexperienced surgeons. The tip of the balloon dilator can be identified as a high echo point moving along the guidewire; thus the location of the dilator can be easily identified and balloon inflation can also be monitored under ultrasound as shown in Fig. 1.

There are some important steps when performing BD under ultrasound guidance. First, a perfect puncture on the most convex point of the renal calyx is very important; prior to BD, we always use a fascia dilator to pre-dilate the tract to 6-Fr which can facilitate the insertion of the balloon dilator. Second, the indwelling length of the balloon is measured and marked, which is equal to the fascia dilator. Third, establishing access to the target renal calyx in cases without hydronephrosis is sometimes difficult because of the limited space between the renal calyx and the stone. In the X-ray guided PCNL, the safety guidewire is usually navigated down to the bladder, which may help to immobilized the kidney when dilation was performed; however, it is quite difficult to pass the guidewire down to the ureter under ultrasound. With its cone-shaped tip, the tip of the balloon dilator can be pushed backward and further away from the collecting system by inflating the balloon, as we observed in some of our cases. Moreover, the end of the balloon dilator is taper-shaped and has an approximate tip length of 0.5 cm beyond the taper; so the measured balloon length should exclude the length of the tip to avoid short dilation in patients without hydronephrosis. Pakmanesh H [25] performed a prospective study to check the feasibility of performing USG Guided Balloon dilatation (24 FR) of PCNL tract for access, and they concluded that a higher rate of short dilation occurred in Amplatz dilation compared that with balloon dilation, which were quite different from present study. A poorly dilated calyx was not good for performing ultrasound-guided tract dilation using a balloon dilator. In case of no hydronephrosis, saline is injected through the ureteral catheter to help the ballooning of the renal calyx. These measures compensate for aiming the target point of renal calyx without the help of X-ray to some extent. Although the success rate of dilation by first attempt was not significant different between the groups, the success rate in patients without hydronephrosis seemed to be lower in the BD group.

This result was quite different from the previous studies [24, 25], we do not suggest BD in cases without hydronephrosis for beginners; for such cases, two-step TMD under ultrasound as we previously reported or x-ray guidance may be a better choice. The cost of the disposable balloon is much higher than that of the TMD in China due to insurance policy; the telescopic metal dilator can be reused, which further lowers the cost of the operation. The cost was full hospital stay, including operation cost(surgical drapes, gowns, gloves, irrigation, lines, lubrication and procedure-specific materials such as hydrophilic wires, access sheath, dilators, laser fibers and baskets),auxiliary procedures cost, cost of treatment for complication. Because there was no significant difference regarding the complication rate, hospital stay and needs for auxiliary treatments between the two groups; the reason for this big difference was that telescopic metal dilator was reusable, which can help to reduce the cost of the operation. Although the operation time was shorter in the BD group, balloon dilator was only for single use, which cost approximately 600 dollar, and this cost was not covered by insurance in this hospital.

There are some limitations in the present study. First, the study reflects the experience of a single center. Surgeons in this hospital were well trained in ultrasound-guided PCNL. Second our inability to find significant difference in some parameters, such as stone-free and complication rates may be secondary to limited patients number.

## Conclusion

In conclusion, PCNL using BD under totally ultrasound guidance is feasible, BD has acceptable complication and stone free rates; however, BD under ultrasound is not suggested for stone cases without hydronephrosis.

## Data Availability

Supporting data can be accessed via the hospital database by contacting the corresponding author upon request.

## Abbreviations

BD: Balloon dilation
PCNL: Percutaneous nephrolithotomy
TMD: Telescopic metal dilation
CT: Computed Tomography
BMI: Body mass index
CIRF: Clinically insignificant residual fragments

## References

[1] Fernstrom I, Johansson B (1976). Percutaneous pyelolithotomy. A new extraction technique. Scand J Urol Nephrol.

[2] Preminger GM, Assimos DG, Lingeman JE, Nakada SY, Pearle MS, Wolf JS Jr. Chapter 1: AUA guideline on management of staghorn calculi: diagnosis and treatment recommendations. J Urol. 2005;173:1991–2000. 10.1097/01.ju.0000161171.67806.2a 15879803

[3] Ozok HU, Sagnak L, Senturk AB, et al. A comparison of metal telescopic dilators and Amplatz dilators for nephrostomy tract dilation in percutaneous Nephrolithotomy[J]. J Endourol. 2011;76:1–5. 10.1089/end.2011.0291 21999400

[4] Dehong C, Liangren L, Huawei L, Qiang W. A comparison among four tract dilation methods of percutaneous nephrolithotomy: a systematic review and meta-analysis. Urolithiasis. 2013;41:523–30. 10.1007/s00240-013-0598-z 23975408

[5] Gonen M, Istanbulluoglu OM, Cicek T, et al. Balloon dilatation versus Amplatz dilatation for nephrostomy tract dilatation [J]. J Endourol. 2008;22:901–4. 10.1089/end.2007.0167 18429681

[6] Frattini A, Barbieri A, Salsi P, Sebastio N, Ferretti S, Bergamaschi E, et al. One shot: a novel method to dilate the nephrostomy access for percutaneous lithotripsy. J Endourol. 2001;15:919–23. 10.1089/089277901753284143 11769847

[7] Kukreja R, Desai M, Patel S, Bapat S. Factors affecting blood loss during percutaneous nephrolithotomy: prospective study. J Endourol. 2004;18:715–22. 10.1089/end.2004.18.715 15659890

[8] Karami H, Rezaei A, Mohammadhosseini M, Javanmard B, Mazloomfard M, Lotfi B. Ultrasonography-guided percutaneous nephrolithotomy in the flank position versus fluoroscopyguided percutaneous nephrolithotomy in the prone position: a comparative study. J Endourol. 2010;24:1357–61. 10.1089/end.2009.0099 20618100

[9] Karami H, Arbab AHMM, Rezaei A, Mohammadhoseini M, Rezaei I. Percutaneous nephrolithotomy with ultrasonographyguided renal access in the lateral decubitus flank position. J Endourol. 2009;23:33–6. 10.1089/end.2008.0433 19178170

[10] Falahatkar S, Neiroomand H, Enshaei A, Kazemzadeh M, Allahkhah A, Jalili MF. Totally ultrasound versus fluoroscopically guided complete supine percutaneous nephrolithotripsy: a first report. J Endourol. 2010;24:1421–6. 10.1089/end.2009.0407 20687858

[11] Yan S, Xiang F, Yongsheng S. Percutaneous nephrolithotomy guided solely by ultrasonography: a 5-year study of >700 cases. BJU Int. 2013;112:965–71. 10.1111/bju.12248 23889729

[12] Thomas K, Smith NC, Hegarty N, Glass JM. The Guy’s stone score—grading the complexity of percutaneous Nephrolithotomy procedures. UROLOGY. 2011;78:277–81. 10.1016/j.urology.2010.12.026 21333334

[13] Dindo D, Demartines N, Clavien PA. Classification of surgical complications: a new proposal with evaluation in a cohort of 6336 patients and results of a survey. Ann Surg. 2004;240:205–13. 10.1097/01.sla.0000133083.54934.ae PMC1360123 15273542

[14] Hellawell GO, Mutch SJ, Thevendran G, Wells E, Morgan RJ. Radiation exposure and the urologist: what are the risks? J Urol. 2005;174:948–52. 10.1097/01.ju.0000170232.58930.8f 16094003

[15] Torrecilla Ortiz C, Meza Martinez AI, Vicens Morton AJ, et al. Obesity in percutaneous nephrolithotomy. Is body mass index really important? Urology. 2014;84:538–43. 10.1016/j.urology.2014.03.062 25168529

[16] Usawachintachit M, Masic S, Chang HC, Allen IE, Chi T. Ultrasound guidance to assist percutaneous Nephrolithotomy reduces radiation exposure in obese patients. urology. 2016;98:32–8. 10.1016/j.urology.2016.04.012 PMC5648533 27112513

[17] Agarwal M, Agrawal MS, Jaiswal A, Kumar D, Yadav H, Lavania P. Safety and efficacy of ultrasonography as an adjunct to fluoroscopy for renal access in percutaneous nephrolithotomy (PCNL). BJU Int. 2011;108:1346–9. 10.1111/j.1464-410X.2010.10002.x 21251187

[18] Lopes T, Sangam K, Alken P, Barroilhet BS, Saussine C, Shi L, et al. Clinical research Office of the Endourological Society Percutaneous Nephrolithotomy Study Group. The clinical research Office of the Endourological Society Percutaneous Nephrolithotomy Global Study: tract dilation comparisons in 5537 patients. J Endourol. 2011;25:755–62. 10.1089/end.2010.0488 21388242

[19] Tomaszewski JJ, Smaldone MC, Schuster T, Jackman SV, Averch TD. Factors affecting blood loss during percutaneous nephrolithotomy using balloon dilation in a large contemporary series. J Endourol. 2010;24:207–11. 10.1089/end.2009.0402 20039798

[20] Wezel F, Mamoulakis C, Rioja J, Michel MS, de la Rosette J, Alken P. Two contemporary series of percutaneous tract dilation for percutaneous nephrolithotomy. J Endourol. 2009;23:1655–61. 10.1089/end.2009.0213 19558265

[21] Joel AB, Rubenstein JN, Hsieh MH, et al. Failed percutaneous balloon dilation for renal access: incidence and risk factors [J]. Urology. 2005;66:29–32. 10.1016/j.urology.2005.02.018 15992884

[22] Osman M, Wendt-Nordahl G, Heger K, Michel MS, Alken P, Knoll T. Percutaneous nephrolithotomy with ultrasonography-guided renal access: experience from over 300 cases. BJU Int. 2005;965:875–8. 10.1111/j.1464-410X.2005.05749.x 16153221

[23] Ren MH, Zhang C, Fu WJ, et al. Balloon dilation versus Amplatz dilation during ultrasound-guided percutaneous nephrolithotomy for staghorn stones. Chin Med J (Engl). 2014;127:1057–61. 24622434

[24] Zhou T, Chen G, Gao X, et al. X-ray’-free balloon dilation for totally ultrasound-guided percutaneous nephrolithotomy. Urolithiasis. 2015;43:189–95. 10.1007/s00240-015-0755-7 25655249

[25] Pakmanesh H, Daneshpajooh A, Mirzaei M, et al. Amplatz versus balloon for tract dilation in Ultrasonographically guided percutaneous Nephrolithotomy: a randomized clinical trial. Biomed Res Int. 2019;3:3428123. 10.1155/2019/3428123. 10.1155/2019/3428123 PMC6335701 30719442

